# An Invited Reply to: A Comment on: 'The swim-and-sink behaviour of copepods: a revisit to mechanical power requirement and a new hypothesis on function' (2023), by Jiang

**DOI:** 10.1098/rsos.231753

**Published:** 2024-01-03

**Authors:** Houshuo Jiang, J. Rudi Strickler

**Affiliations:** ^1^ Applied Ocean Physics and Engineering Department, Woods Hole Oceanographic Institution, Woods Hole, MA 02543, USA; ^2^ School of Freshwater Sciences, University of Wisconsin-Milwaukee, Milwaukee, WI 53204, USA

**Keywords:** hop-and-sink, swim-and-sink, copepods

We reply to the comments of Weihs [1] on the recent paper by Jiang [2].

Weihs [1] mentioned that the swim-and-sink behaviour of copepods was better known as the hop-and-sink behaviour. The biological reality, however, is that the two behaviours are different. Bainbridge [3] referred to the swimming of the calanoid copepod *Calanus finmarchicus* as ‘hop and sink’ but provided no further description of the behaviour. Later on, Strickler [4] used ‘hop and sink’ to describe the jerky swimming pattern displayed by cyclopoid copepods and provided a detail description and kinematic analysis of the behaviour. Here, we present one of his high-speed Schlieren videos of a cyclopoid copepod in hop-and-sink together with its path speed as a function of time (electronic supplementary material, video S1; figure 1). Each hop starts with the cyclopoid beating its two antennules (A1) and culminates with the cyclopoid sequentially beating its four pairs of swimming legs along with its urosome [5]. Each hop was brief and highly unsteady, with a power stroke duration of 16.9 **±** 0.8 ms (mean **±** s.d., *n* = 6) and attaining a maximum speed of 27.0 **±** 4.1 mm s**^−^**^1^ (mean **±** s.d., *n* = 6). To characterize the briefness or impulsiveness of a hop, we calculate the non-dimensional jump number Jn [6] defined as the ratio of the power stroke duration *τ* to the viscous timescale $L_{p}^{2}/(4\nu )$, where *L*_p_ is the prosome length of the cyclopoid and *ν* is the kinematic viscosity of water. For *τ* = 16.9 ms, *L*_p_ = 0.77 mm, and *ν* = 1.0 × 10**^−^**^6^ m^2^ s**^−^**^1^, Jn = 0.11, indicating that the power stroke duration is much shorter than the viscous timescale. By contrast, the swim-and-sink behaviour of the calanoid copepod *Centropages* sp. involves only the calanoid's cephalic appendages that vibrate continuously during each swimming phase, operating at a much longer upward-swimming duration and attaining a much slower speed (table 2 in [2]). If we neglect the small oscillations due to vibrating of the cephalic appendages and the feeding activities of the calanoid, we can consider swim-and-sink as a piecewise quasi-steady behaviour (electronic supplementary material, video S2; figure 2). Additionally, we should say that the ‘hop and sink’ of the calanoid *C. finmarchicus* seen in the diving observations with the naked eye should actually be ‘swim and sink’.

**Figure 1.**
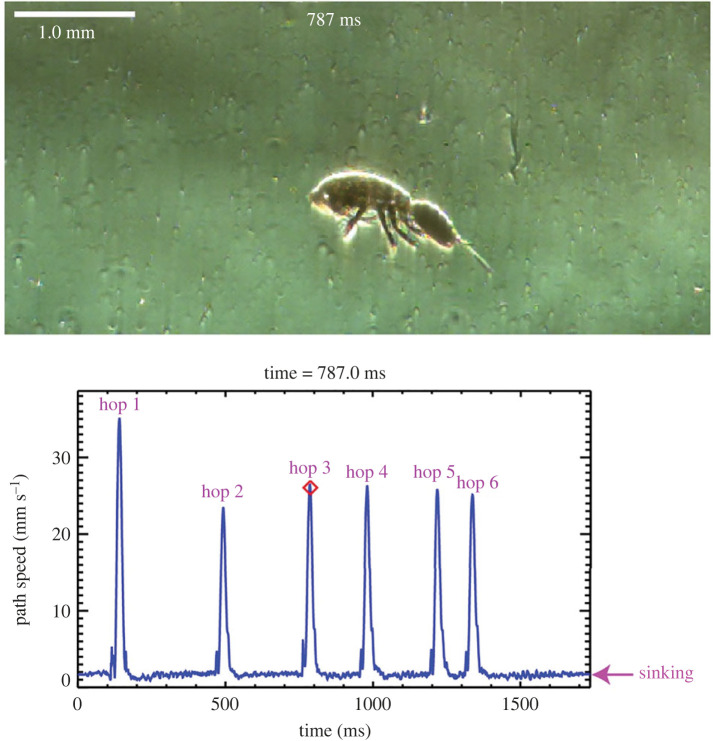
Upper panel: An image frame extracted from a high-speed Schlieren video of a female cyclopoid copepod in hop-and-sink (electronic supplementary material, video S1). Lower panel: the path speed of the cyclopoid as a function of time.

**Figure 2.**
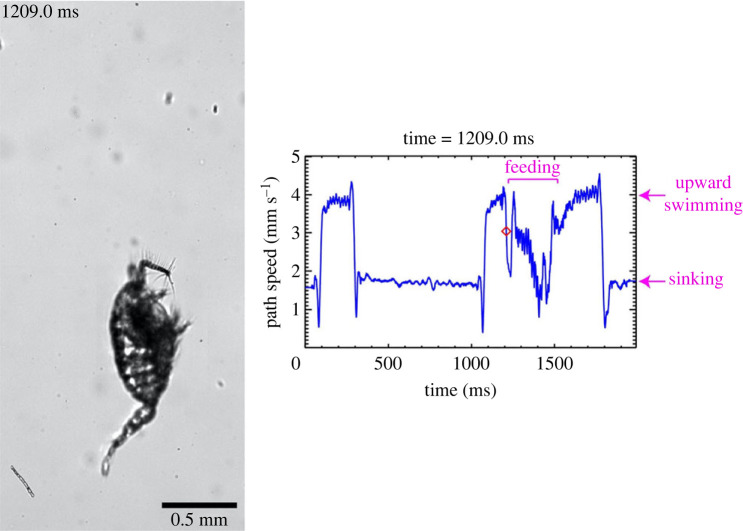
Left panel: an image frame extracted from a high-speed video of a late copepodite of the calanoid copepod *Centropages* sp. performing the swim-and-sink behaviour for detecting, manoeuvring, and capturing a diatom chain (electronic supplementary material, video S2). Right panel: the path speed of the calanoid as a function of time.

Once we distinguish ‘swim and sink’ from ‘hop and sink’, it makes sense that a computational fluid dynamics (CFD) model of a copepod swimming upward at a constant speed was used for an energetic analysis of swim-and-sink considered as a piecewise quasi-steady behaviour [2]. Thus, the conclusion should also make sense, i.e. ‘upward swim-and-sink about a fixed depth always requires more mechanical power than hovering does'. Although they used the term 'hop and sink’ to refer to the copepod behaviour considered in their paper, what Haury & Weihs [7] really considered was the swim-and-sink behaviour (p. 405 in [8]). Both Haury & Weihs [7] and Jiang [2] used the same piecewise quasi-steady assumption; however, the difference is that Haury & Weihs [7] treated a free-swimming copepod as a towed body while Jiang [2] treating a free-swimming copepod as a self-propelled swimming body. It is this difference that has led to two completely different conclusions. On the other hand, an energetic analysis of the true hop-and-sink behaviour—neither Haury & Weihs [7] nor Jiang [2] considered it—does require an unsteady CFD model of copepod jumping, which is currently available (e.g. [9]).

Regardless of ‘swim and sink’ or ‘hop and sink’, the ecological benefit that a copepod gains from performing the behaviour must well repay the associated mechanical energy cost. The ecological functions of the swim-and-sink behaviour of the calanoid copepod *Centropages* sp. have been discussed in [2]. Here, we only briefly discuss the ecological functions of the hop-and-sink behaviour of some cyclopoid copepods. The hop-and-sink behaviour may function as a sensory matched filter [10,11] that aids the cyclopoid in hunting for motile prey or finding mates. The regularly repeated hop-and-sink pattern sets up a hydrodynamic environment that is the same from hop to hop; once a prey runs into the scene and disturbs the environment, the cyclopoid perceives the prey by comparing the disturbed environment with the normal environment (the filter), immediately breaks from the hop-and-sink pattern, and switches to a hunting action [12]. During mate finding and courtship, a male cyclopoid was observed to perform a hop-and-sink swimming to follow, in a remarkable synchronization, the female that was 2–3 mm ahead and also in hop-and-sink [13]. Additionally, there is the possibility that hop-and-sink is a quiet swimming mode because each hop generates a spatially limited, temporally fast-decaying viscous vortex ring, similar to what has been shown for swimming by jumping in copepods [14], thereby reducing predation risk from flow-sensing predators.

## Data Availability

Supplementary material is available online [15].
